# *Hypericum lanceolatum *Lam. Medicinal Plant: Potential Toxicity and Therapeutic Effects Based on a Zebrafish Model

**DOI:** 10.3389/fphar.2022.832928

**Published:** 2022-03-11

**Authors:** Laura Gence, Danielle Fernezelian, Matthieu Bringart, Bryan Veeren, Armelle Christophe, François Brion, Olivier Meilhac, Jean-Loup Bascands, Nicolas Diotel

**Affiliations:** ^1^ Université de La Réunion, INSERM, Diabéte athérothrombose Thérapies Réunion Océan Indien (DéTROI), Saint-Denis de La Réunion, France; ^2^ Unité D’Écotoxicologie des Substances et des Milieux (ESMI), Institut National de L’Environnement Industriel et des Risques (INERIS), Verneuil-en-Halatte, France; ^3^ CHU de La Réunion, Saint-Denis, France

**Keywords:** diabetes, LC MS/MS, neurogenesis, obesity, regeneration, Toxicity, Zebrafish

## Abstract

*Hypericum lanceolatum* Lam. (*H. lanceolatum*) is a traditional medicinal plant from Reunion Island used for its pleiotropic effects mainly related to its antioxidant activity. The present work aimed to 1) determine the potential toxicity of the plant aqueous extract *in vivo* and 2) investigate its putative biological properties using several zebrafish models of oxidative stress, regeneration, estrogenicity, neurogenesis and metabolic disorders. First, we characterized the polyphenolic composition by liquid chromatography-tandem mass spectrometry (LC-MS/MS) and identified chlorogenic acid isomers, quercetin and kaempferol derivatives as the major compounds. We then evaluated for the first time the toxicity of an aqueous extract of *H. lanceolatum* and determined a maximum non-toxic concentration (MNTC) in zebrafish eleutheroembryos from 0 to 96 hpf following OECD (Organization for Economic Cooperation and Development) guidelines. This MNTC test was also determined on hatched eleutheroembryos after 2 days of treatment (from 3 to 5 dpf). In our study, the anti-estrogenic effects of *H. lanceolatum* are supported by the data from the EASZY assay. In a tail amputation model, we showed that *H. lanceolatum* at its MNTC displays antioxidant properties, favors immune cell recruitment and tissue regeneration. Our results also highlighted its beneficial effects in metabolic disorders. Indeed, *H. lanceolatum* efficiently reduces lipid accumulation and body mass index in overfed larva- and adult-models, respectively. In addition, we show that *H. lanceolatum* did not improve fasting blood glucose levels in a hyperglycemic zebrafish model but surprisingly inhibited neurogenesis impairment observed in diabetic conditions. In conclusion, our study highlights the antioxidant, pro-regenerative, anti-lipid accumulation and pro-neurogenic effects of *H. lanceolatum in vivo* and supports the use of this traditional medicinal plant as a potential alternative in the prevention and/or treatment of metabolic disorders.

## Introduction

For decades, traditional herbal medicine has been used worldwide against a multitude of disorders and diseases. In the Mascarene Islands (Reunion, Mauritius, and Rodrigues), some plants are still used by local people as beverages or external treatments to treat minor infections of skin, gastrointestinal or urinary tracts, ulcers, infertility, epilepsy as well as chronic diseases including obesity and diabetes (Gurib-Fakim and Brendler, 2004). Among these territories of the Indian Ocean, Reunion Island is known for the richness and diversity of its plant biodiversity which offers a large number of bioactive molecules contributing to the health benefits of many plants. As well, the International Union for Conservation of Nature (IUCN, 2013) classified the island biodiversity among the 34 global biodiversity hotspots. More than 1700 species have been identified with about 237 listed endemic plants (Reunion’s Regional Tourism Committee, 2008). Among these plants, 27 have recently been registered in the French Pharmacopeia because of their antioxidant, hypolipidemic, anti-inflammatory or healing properties in traditional medicine particularly alleged in traditional medicine (Aplamedom Reunion, 2021; Lavergne, 2001; Agence Nationale De Sécurité Du Médicament Et Des Produits De Santé, 2021).

Among these medicinal plants, *Hypericum lanceolatum* Lam. (*H. lanceolatum*) that belongs to Hypericaceae family, is an indigenous plant that is mainly recommended by local herbal tea makers as a good anti-inflammatory treatment in case of gastrointestinal disorders. It has also been suggested that the plant could be used against diabetes or could promote blood detoxification and act against fatigue associated with chronic diseases (Lavergne, 2001; Smadja and Marodon, 2016). Despite these traditional uses, scientific data about the toxicity of *H. lanceolatum* and evidence regarding its real health benefits are very scarce. An *in vivo* study showed that a *H. lanceolatum* hydroalcoholic extract, orally administrated for 14 days, was non-toxic in rats at a concentration of 2 g/kg (Brillant et al., 2006). In addition, some studies, performed *in vitro*, demonstrated the therapeutic potential of *H. lanceolatum* against diverse infectious diseases including malaria or leishmaniasis (Zofou et al., 2011; Kowa et al., 2019) as well as in a model of oxidative stress-induced erythrocyte hemolysis or preadipocytes necrosis (Checkouri et al., 2020).

According to their traditional uses, the leaves and stems are usually boiled in water for the preparation of herbal teas. This allows the extraction of many molecules including polyphenols and polycyclic polyprenylated acylphloroglucinols (PPAPS), which are thought to be associated with the potential beneficial effects of the plant (Checkouri et al., 2020). Previous phytochemical reports on *H. lanceolatum* revealed the presence of several polyphenolic species (flavonoids, xanthones, benzophenones, xanthonolignoids) in the leaves and stem barks but also the presence of terpenoids, anthraquinones and PPAPS derivatives (Wabo et al., 2012; Fobofou et al., 2016; Checkouri et al., 2020). Therefore, considering its traditional uses and composition, *H. lanceolatum* appears to have therapeutic virtues to counteract some adverse effects induced by metabolic disorders. It is then very important to perform rigorous studies to determine potential toxicity and provide evidence of the putative therapeutic effects of this medicinal plant.

To this end, we used the zebrafish (*Danio rerio*) model allowing to study inflammation, oxidative stress and regeneration (MacRae and Peterson, 2015; Lam and Peterson, 2019). Zebrafish share a strong genomic homology with humans (>70%) and display many physiological similarities with mammals, notably during embryonic development (Howe et al., 2013; MacRae and Peterson, 2015). For these reasons, specific tests have been established and approved by the Organization for Economic Co-operation and Development (OECD) for assessing toxicity during zebrafish development (OCDE, 2013). In addition, zebrafish exhibit common pathophysiological processes with mammalian metabolic disorders (Williams and Hong, 2011; Howe et al., 2013) and many zebrafish models have been developed in larvae and adults to study diabetes and obesity (Zang et al., 2018).

Thus, the present study was designed to determine the composition of the aqueous extract of *H. lanceolatum*, its *in vivo* potential acute toxicity in zebrafish and to screen its biological properties. To this end, we first analyzed the polyphenolic composition of the plant aqueous extract by liquid chromatography-tandem mass spectrometry (LC-MS/MS) and characterized its maximum non-toxic concentration during zebrafish development (embryo and eleutheroembryo) by evaluating morphological and behavioral parameters and toxicity biomarkers. Next, using reliable zebrafish models, we investigated the effects of the aqueous extract of *H. lanceolatum* on oxidative stress, inflammation, tissue regeneration and also on metabolic complications including lipid accumulation, weight gain, hyperglycemia and its impact on neurogenesis.

## Materials and Methods

### Plant Material and Infusion Preparation (Aqueous Extract)

Dried leaves of *Hypericum lanceolatum subsp. lanceolatum* (*H. lanceolatum*) (Grand Coude, Saint-Joseph, Reunion Island - Reference: FJBMCA160825AA) were purchased from the agricultural cooperative CAHEB (Coopérative Agricole des Huiles Essentielles de Bourbon, Le Tampon, Reunion Island). Next, the dried plants were crushed with a laboratory grinder (Retsch) and stored at –20°C until use. To obtain the infusion, crushed leaves (1 g) were incubated with 50 ml of boiling zebrafish water or E3 embryo medium (300 mM NaCl, 10 mM KCl, 20 mM CaCl_2_, 50 mM MgCl_2_, pH 7.2) for 10 min under stirring, at room temperature (RT) as previously described (Ghaddar et al., 2020). The aqueous extract was then filtered and processed for further dilutions.

### Total Polyphenol Content

The total polyphenol content of the aqueous extract was determined by the Folin-Ciocalteu assay adapted from Septembre-Malaterre et al. (2016). In a 96-well microplate, 25 µL of the aqueous extract were incubated with 125 µL of diluted Folin-Ciocalteu’s reagent and 100 µL of sodium carbonate (0.7 M) followed by incubation at 50°C for 5 min. Then, the microplate was cooled at 4°C for 5 min and the absorbance was measured at 760 nm (FLUOstar Omega, Bmg Labtech). Gallic acid solution was used for the standard calibration curve and results are expressed as mg gallic acid equivalent (GAE) per g of dried plant.

### *In vitro* DPPH Radical Scavenging Activity

The chemical antioxidant capacity of the extract was assessed according to the DPPH (2,2-diphenyl-1-picrylhydrazyl) method described by Delveaux et al. (2020), with minor modifications. In a 96-well microplate, 40 µL of the aqueous extract or water (Blank) were incubated with 200 µL of a DPPH solution (0.25 mM in methanol) at 30°C for 25 min. The absorbance was measured at 517 nm (FLUOstar Omega, Bmg Labtech). Ascorbic acid was used as positive control. The antioxidant capacity was calculated as: % antioxidant capacity = [(Abs_Blank_—Abs_Sample_)/Abs_Blank_] x100. Results are indicated in “IC_50_-like” values (concentration leading to 50% reduction in chemical antioxidant capacity and calculated using a non-linear regression analysis), in g/L. Note that this chemical test was done to check for potential antioxidant compounds but is of no pharmacological relevance.

### Polyphenol Identification and Quantification (LC-MS/MS)

Polyphenols were identified by ultra-high-performance liquid chromatography, coupled with diode array detection and HESI-Orbitrap mass spectrometer (Q-Exactive™ Plus, Thermo Scientific) as previously described (Veeren et al., 2020). Briefly, 10 µL of the sample were injected using an UHPLC system equipped with a Thermo Fisher Ultimate 3,000 series WPS-3000 RS autosampler and then separated on a PFP column (2.6 μm, 100 mm × 2.1 mm, Phenomenex, Torrance, CA, United States). The column was eluted with a gradient mixture of 0.1% formic acid in water (A) and 0.1% formic acid in acetonitrile (B) at the flow rate of 0.450 ml/min, with 5% B at 0.00–0.1 min, 35% B at 0.1–7.1 min, 95% B at 7.2–7.9 min, and 5% B at 8.0–10 min. The column temperature was held at 30°C and the detection wavelength was set to 280 nm, allowing the identification of polyphenols.

For the mass spectrometer conditions, a Heated Electrospray Ionization source II (HESI II) was used. Nitrogen was used as the drying gas. The mass spectrometer conditions were optimized as follows: spray voltage 2.8 kV, capillary temperature 350°C, sheath gas flow rate 60 units, aux gas flow rate 20 units, and S lens RF level 50. Mass spectra were registered in full scan mode from *m*/*z* 100 to 1,500 in negative ion mode at a resolving power of 70,000 FWHM at *m*/*z* 400. The automatic gain control (AGC) was set at 1e6. The MS/MS spectra were obtained by applying a relative higher energy collisional dissociation (HCD) energy of 25%. The identification of the compounds of interest was based on their retention time, accurate mass, elemental composition, MS fragmentation pattern, and comparisons with available standards and the advanced mass spectral database, *m*/*z* Cloud, https://www.mzcloud.org. Data were acquired with the XCalibur 4.2 software (Thermo Fisher Scientific Inc.) and processed with the compound discoverer 2.1 and the Skyline 20.1 software (MacCoss Lab.).

For quantification, standard stock solutions of caffeic acid, caffeoylquinic acid, kaempferol, quercetin, protocatechuic acid and gallic acid (Sigma Aldrich, St. Louis, MO, United States) were dissolved in methanol at a concentration of 1 mg/mL. A mixed stock solution containing 10 μg/ml of each polyphenol standard was prepared in methanol and then diluted in 0.1% formic acid to obtain the desired calibration curves ranging from 10 to 4,000 ng/ml. After linear regression calculation, the analyte concentrations were determined and expressed in mg/g of dried plant (calibration curves of each standard polyphenol had a correlation coefficient (R2) of 0.99).

### Animals and Ethics

Zebrafish (*Danio rerio*) were housed in the zebrafish facility at CYROI/DéTROI, La Réunion (A974001). Adult wild-type (WT) and transgenic tg(*GFAP::GFP*) zebrafish (AB strain), aged 3–6 months, were maintained under standard conditions of temperature (28.5°C for adult fish and 26.5°C for embryos/eleutheroembryos/larvae), photoperiod (14 h dark and 10 h light), pH (7.4) and conductivity (400 µS). Fish were fed every day (3 times a day) with commercial dry food (Gemma Micro 300, Skretting, France). All animal experiments were conducted in accordance with the French and European Community Guidelines for the Use of Animals in Research (86/609/EEC and 2010/63/EU) and approved by the local Ethics Committee for animal experimentation of CYROI and the French Government (APAFIS#29570-2020092910327075 v3; APAFIS#2021072814123963 v4; APAFIS#2021080209405969_v8).

Note that in this manuscript, the term “eleutheroembryo” refers to a developmental stage from 0 to 120 h post-fertilization (hpf). The term “larva” is used when the animal begins to feed autonomously, from approximately 5-6-dpf.

### Fish Embryo Acute Toxicity and Eleutheroembryo Toxicity Tests

The FET test was performed using the OECD guideline 236 (OCDE, 2013). After breeding, fertilized eggs were selected (<3-hpf) and transferred in a 24-well plate containing freshly prepared *H. lanceolatum* infusion at different concentrations (5, 2.5, 1.25, 0.625, 0.3125, and 0.156 g/L) or E3 embryo medium as control condition. A total of 20 embryos were used for each condition (5 embryos in 1 ml/well) and every solution was changed each day up to 96-hpf. The embryos were daily analyzed, under a microscope, for evaluation of coagulation, somite formation, tail detachment and heartbeats as indicators of lethality and/or abnormal development. In addition, some morphological malformations (spinal curvature, pericardial edema, pigmentation, delayed development) and hatching were also considered until the end of the experiments.

Since morphogenesis is nearly complete at 3-dpf, toxicity was assessed on eleutheroembryos from 3- to 5-dpf, according to the same OECD procedure. Briefly, fertilized eggs were allowed to grow normally at 26.5°C, in fish water. At 3-dpf, eleutheroembryos were incubated with the different solutions (*H. lanceolatum* extract at the same concentrations or E3 medium) in a 24-well plate. A total of 20 eleutheroembryos were used for each concentration (5 eleutheroembryos in 1 ml/well) and each solution was changed daily until 5-dpf. The eleutheroembryos were evaluated daily for mortality and morphological malformations. In both embryo and eleutheroembryo experiments, the Maximum Non-Toxic Concentration (MNTC) corresponds to the highest concentration that did not cause death.

### RNA Extraction, Reverse Transcription, qPCR and tg(*GFAP::GFP*) Fluorescence Analysis

Transgenic tg(*GFAP::GFP*) eleutheroembryos were treated from 3- to 5-dpf with E3 medium or aqueous extract of *H. lanceolatum* at the Maximum Non-Toxic Concentration (MNTC, 0.3125 g/L) and analyzed for the expression of genes recognized as biomarkers of kidney (*ctgf*), liver (*fabp10a*, *gclc*) and heart (*erg*) damage and the *fkbp5* gene modulated by glucocorticoids. After a 48 h-incubation period, eleutheroembryos were pooled and immediately frozen at −20°C for at least 2 h, prior to RNA extraction.

For RNA extraction, pooled eleutheroembryos (n = 40) were homogenized using a Tissue Lyser II (Qiagen). Total RNA was extracted using the RNeasy^®^ mini kit (Qiagen), following the manufacturer’s instructions. Extracted RNA was then quantified with a UVS-99 (ACTGene) and all samples were stored at −80°C. For reverse transcription, 2 µg of RNA were reverse transcribed to cDNA using random hexamers (Jena Bioscience) and NxGen^®^ MMLV reverse transcriptase (Lucigen). Next, qPCR experiment was performed using the CFX Connect™ Real-Time System (Bio-Rad) and BrightGreen Express 2X qPCR MasterMix-ROX (Abm). Specific zebrafish primers were used (Table 1) and the relative gene expression was normalized to the expression of *ef1α* reference gene. Changes in gene expression were calculated using the 2^−ΔΔCt^ method.

**TABLE 1:** qPCR primer sequences.

Zebrafish gene	Gene name	Marker of	Primer sequences
*fapb10a*	*fatty acid binding protein 10a*	Hepatotoxicity	F: CCA​GTG​ACA​GAA​ATC​CAG​CA
			R: GTT​CTG​CAG​ACC​AGC​TTT​CC
*gclc*	*glutamate cysteine ligase catalytic subunit*	Hepatotoxicity	F: AAA​ATG​TCC​GGA​ACT​GAT​CG
			R: AAC​GTT​TCC​ATT​TTC​GTT​GC
*erg*	*ether-a-go-go related gene*	Cardiotoxicity	F: CAG​ATG​CTC​CGT​GTG​AAA​GA
			R: TGC​GGT​TCA​GAT​GAA​GAC​AG
*ctgg*	*connective tissue growth factor*	Nephrotoxicity	F: CTC​CCC​AAG​TAA​CCG​TCG​TA
			R: TCC​ACC​AAA​CAC​ACA​AGT​GG
*fkbp5*	*FK506 binding protein 5*	Glucocorticoid signaling	F: CAA​AAG​GGG​GAA​TGC​TGT​T
			R: TTC​TTT​TCT​GCC​CTC​TTT​GC
*ef1α*	*elongation factor 1-alpha*	Housekeeping gene	F: AGC​AGC​AGC​TGA​GGA​GTG​AT
			R: CCG​CAT​TTG​TAG​ATC​AGA​TGG

In parallel, transgenic eleutheroembryos were used to determine a potential neurotoxic effect of the plant extract by measuring the GFAP::GFP protein fluorescence. Briefly, 3-dpf eleutheroembryos were similarly treated with E3 medium or the aqueous extract at the MNTC (0.3125 g/L), for 2 days. Then, eleutheroembryos were fixed in 4% paraformaldehyde (PFA) in phosphate-buffered saline (PBS), overnight at 4°C, and examined under fluorescence microscope.

### Detection of Endocrine Active Substances, Acting Through Estrogen Receptors, Using Transgenic tg*(cyp19a1b:GFP)* Zebrafish EmbrYos (EASZY Test)

For evaluating the potential estrogenic activity of *H. lanceolatum*, the EASZY assay was conducted according to the OECD TG 250 (OCDE, 2021). Newly fertilized *cyp19a1b*-GFP eleutheroembryos were transferred to glass crystallizers and exposed from 0- to 4-dpf in an incubator (28 ± 1°C) under semi-static conditions with a total renewal of the medium each day. Each condition consisted in a triplicate of crystallizers containing 7 embryos and 15 ml of water. Eleutheroembryos were exposed to a range of non-lethal concentrations of *H. lanceolatum* extract (from 0.3125 to 0.0195 g/L) and compared to negative control (breeding water) and positive control (17α ethinylestradiol EE2 0.05 nM). At the end of exposure, transgenic zebrafish eleutheroembryos were collected for fluorescence imaging. The analysis was done on a total of 26–38 eleutheroembryos per test condition in two independent experiments. In a third assay, eleutheroembryos were exposed to E2 (2 and 10 nM) alone or in co-exposure with *H. lanceolatum* at a concentration of 0.156 g/L. To validate each test, the compliance with the validity criteria have been checked.

### Behavioral Analysis

The locomotor activity of larvae was performed on 6-dpf animals, given that a previous study recommended the use of 6- and 7-dpf larvae for better assessment of locomotion (Colwill and Creton, 2011). Consequently, 3-dpf larvae were incubated in 1 ml of E3 medium (Control) or aqueous extract of *H. lanceolatum* at the MNTC (0.3125 g/L), in a 24-well plate (1 larva/well), for 3 days at 26.5°C. The treatment was renewed every day. At 6-dpf, locomotion was monitored using the Viewpoint Zebrabox system (Viewpoint Behavior Technology), after an adaptation period of 10 min. Then, inactivity (<3 mm/s), small activity (3–6 mm/s) and large activity (>6 mm/s) were recorded for 10 min, in the dark. The analysis was done on a total of 12 larvae.

### Caudal Fin Amputation

Adult zebrafish (3–6 months old) and 3-dpf eleutheroembryos were anesthetized with 0.02% tricaine (MS-222). Then, caudal fin amputations were performed using a scalpel. For adult fish, we performed the amputation at 50% of total caudal fin. Concerning eleutheroembryos, transection was done near the notochord (without injuring the notochord), under a stereomicroscope.

Caudal fin amputations were performed for oxidative stress, immune cell recruitment and regeneration analyses, as described below.

### Oxidative Stress, Immune Cell Recruitment and Tissue Regeneration Studies

Oxidative stress experiments were performed on adult WT zebrafish (3–6 months old). Prior to amputation, fish were treated with the aqueous extract of *H. lanceolatum* at the MNTC (0.3125 g/L) or fish water, for 4 h. Immediately after caudal fin amputation, fish were incubated with DCFH-DA (2ʹ,7ʹ-Dichlorofluorescein Diacetate, Sigma) (10 µM) and DHE (Dihydroethidium, Sigma) (10 µM) probes, in fish water, for 30 min in the dark. After incubation with the probes, the fish were rinsed and imaged with a stereomicroscope. The experiments were repeated thrice on a total of 9 animals.

For immune cell recruitment experiments, 3-dpf WT eleutheroembryos were used. After injury, eleutheroembryos were incubated in E3 medium or *H. lanceolatum* extract at the MNTC (0.3125 g/L), for 6 h at 26.5°C. At the end of the incubation period, eleutheroembryos were fixed in 4% PFA, overnight at 4°C, and processed for immunohistochemistry analysis using anti-myeloperoxidase (Mpo, see below) and anti-L-Plastin (Lcp1, see below) antibodies. Three independent experiments were performed on a total of 34 eleutheroembryos.

The regeneration procedure was achieved on WT adult fish and eleutheroembryos. After amputation, fish were treated with *H. lanceolatum* aqueous extract at the MNTC (0.3125 g/L) or fish water, for 14 days. Every solution was changed each day. Images were taken using a stereomicroscope, under anesthesia, at 0-, 7- and 14-days post-lesion (dpl). The experiment was done on a total of 7 animals/condition. In a similar way, 3-dpf amputated eleutheroembryos were treated with *H. lanceolatum* aqueous extract at the MNTC (0.3125 g/L) or E3 medium (Control), for 7 days. For dexamethasone exposure, 3-dpf amputated eleutheroembryos were incubated with 0.4% ethanol in E3 medium (Vehicle), 500 µM of dexamethasone (DEX) or dexamethasone and *H. lanceolatum* aqueous extract treatment at 0.3125 g/L (DEX + HL), for 3 days. Every solution was changed each day and images were taken, under anesthesia, at 0 and 3 dpl. Three independent experiments were performed on 20–39 eleutheroembryos/condition. The area of regenerated fins was semi-quantified by using Image J software.

### Chronic Hyperglycemic Model

To induce a hyperglycemic state, adult zebrafish (3–6 months old) were immersed in 111 mM D-Glucose for 14 days, as previously reported (Capiotti et al., 2014; Dorsemans et al., 2017a; Dorsemans et al., 2017b). Briefly, in a 3.5 L-tank, fish were incubated with fish water (Control) or 111 mM of D-Glucose (Chronic hyperglycemia, CHG), for 7 days. At day 7, CHG fish were divided into two groups: an untreated group (CHG) and a *H. lanceolatum* treated group (CHG + HL). CHG + HL fish were treated with *H. lanceolatum* aqueous extract at the MNTC (0.3125 g/L). Solutions were changed twice a day. On day 14, fish were sacrificed and fasting blood glucose levels (12 h-fasting period) were measured using a glucometer (OneTouch^®^ Verio Flex). Then, fish were fixed in 4% PFA, overnight at 4°C, prior to brain dissections and storage at −20°C in 100% methanol (MeOH) for PCNA immunohistochemistry.

### Diet-Induced Overweight/Obesity and High-Fat Diet Models

The induction of overweight/obesity in adult zebrafish (3–6 months old) was carried out according to a previously described protocol (Ghaddar et al., 2020). Adult WT zebrafish were randomly divided into 3 dietary groups, for 2 weeks (with 10 fish/3.5-L tank): normally fed (Control), overfed (DIO) and overfed supplemented with *H. lanceolatum* aqueous extract (DIO + HL). The control group was fed daily with commercial dry food in the morning (15 mg/fish/day) and freshly hatched *Artemia* in the afternoon (6 mg/fish/day). The overfed groups were fed 6 times a day with commercial dry food (52.5 mg/fish/day) and 3 times a day with *Artemia* (60 mg/fish/day). Treatment with *H. lanceolatum* started at day 7, for 1 week: fish were placed overnight in *H. lanceolatum* aqueous extract at the MNTC (0.3125 g/L).

Because larvae begin feeding at approximately 5-dpf and exhibit a fully functional gastrointestinal tract from 7-dpf (Holmberg et al., 2003; Flores and Nguyen, 2020), a HFD model was also developed in larvae. In brief, 7-dpf larvae were divided to 3 groups: normally fed (Control), overfed (HFD) and overfed supplemented with *H. lanceolatum* aqueous extract at the MNTC (HFD + HL). From 7- to 11-dpf, larvae were maintained with the corresponding diet and the solutions were renewed every day. For the control group, larvae were incubated with 0.1% of commercial dry food (Gemma Micro 75, Skretting) diluted in E3 medium. Overfed larvae were placed in 0.1% of egg yolk (Sigma) diluted in E3 medium (HFD) or in *H. lanceolatum* aqueous extract (HFD + HL). At 11-dpf, larvae were anesthetized on ice, fixed in 4% PFA overnight at 4°C, prior to Oil red O (ORO) staining.

### Body Weight, Body Mass Index and Fasting Blood Glucose Measurements

Body weight and length of adult zebrafish (3–6 months old) were assessed at the beginning of the study and every week. Length was measured from the tip of the mouth to the end of the tail. The body mass index (BMI) was calculated by dividing the body weight (kg) with the square of the total length (m^2^). For glycemia measurement, fish were fasted overnight, sacrificed on ice water in order to avoid blood glucose variation due to anesthesia and glycemia was determined using a glucometer.

### Immunohistochemistry

Myeloperoxidase (Mpo), L-Plastin (Lcp1) and Proliferating cell nuclear antigen (PCNA) immunostainings were performed on whole 3-dpf eleutheroembryos (for Mpo and Lcp1) and brain sections (for PCNA).

For Mpo immunostaining, fixed eleutheroembryos were dehydrated in 100% MeOH and stored overnight at −20°C until use. For Lcp1 immunostaining, fixed eleutheroembryos were directly permeabilized with PTw (1X PBS containing 0.1% Tween-20) and treated with cold acetone (−20°C) for 10 min. Then, eleutheroembryos were rehydrated, washed with PTw and blocked with PTw containing 2% BSA and 1% DMSO, for 1 h at RT. Next, eleutheroembryos were incubated with a rabbit anti-zebrafish Mpo antibody (1:200 dilution, Abcam, Reference: ab210563) or a rabbit anti-zebrafish Lcp1 antibody (1:500 dilution, GeneTex, Reference: GTX124420), overnight at 4°C. Next day, eleutheroembryos were rinsed with PTw and incubated with Alexa Fluor^®^ 594 donkey anti-rabbit antibody (1:500 dilution, Abcam, Reference: ab150064) and DAPI (4′,6-diamidino-2-phenylindole, Dihydrochloride) (1 ng/ml, ThermoFisher, Reference: D1306) for 2 h at RT. Then, eleutheroembryos were washed 3 times with PTw for imaging. Pictures were made and analyzed by two different users. Three independent experiments were done on a total of 34 eleutheroembryos/condition.

Brain cell proliferation analysis was realized according to previous protocols (März et al., 2011; Dorsemans et al., 2017a). For that, adult zebrafish brains were rehydrated, permeabilized with PTw and embedded in 2% agarose before performing 50 µm-thick sections using a vibratome (VT1000S, Leica). After 1 h of blocking with PTw containing 0.2% BSA and 1% DMSO, sections were incubated with a mouse anti-PCNA antibody (1:500 dilution, Dako, Reference: M0879) overnight at 4°C. The next day, the sections were washed 3 times with PTw and incubated with Alexa Fluor^®^ 594 goat anti-mouse antibody (1:500 dilution, ThermoFisher, Reference: A11005) and DAPI for 2 h at RT. Finally, the sections were washed with PTw and mounted on slides with Aqua-Poly/Mount (Polysciences).

### Oil Red O Staining

Fixed larvae were rinsed with 1X PBS, dehydrated in 100% MeOH and stained with freshly prepared 0.15% ORO (Sigma) (in 60% isopropanol/1X PBS), overnight at RT. Next, larvae were rehydrated and washed with 1X PBS before imaging.

### Microscopy

Pictures were acquired using a Nikon eclipse 80*i* equipped with a Hamamatsu camera, a Nikon SMZ18 stereomicroscope equipped with a Nikon DS-F*i*3 Camera and the respective NIS elements software.

Images were analyzed using the ImageJ software (https://imagej.nih.gov/ij/). Quantification of regenerated fin area, fluorescence intensity and cell numbers were performed by two different users blinded to the treatment, and then the results were averaged.

### Statistical Analysis

Data were presented as means ± SEM (Standard error of the mean). All statistical analyses were performed using GraphPad Prism 8 software. Comparisons between groups were performed using Student t-test and one-way ANOVA. Values were considered as significantly different when *p*-values < 0.05.

## Results

### Total Polyphenol Content, Phytochemical Composition and Antioxidant Capacity of the Aqueous Extract of *H. lanceolatum*

*H. lanceolatum* herbal tea is widely used in traditional medicine in Reunion Island to reduce inflammation and diabetes, and is described as a source of bioactive molecules including polyphenols (Wabo et al., 2012; Checkouri et al., 2020). For these reasons, an aqueous extract of *H. lanceolatum* was prepared and evaluated for polyphenol quantification using the colorimetric Folin-Ciocalteu method. A total of 103.2 ± 6.1 mg GAE (gallic acid equivalent)/g of dried plant was reported for this infusion (Table 2). Given that polyphenols are well documented to be correlated with antioxidant properties, the free radical scavenging capacity of the *H. lanceolatum* aqueous extract was evaluated *in vitro* by performing the DPPH assay. Although this chemical antioxidant assay is of no pharmacological relevance, it provided an “IC_50_-like” antioxidant capacity estimated to 0.86 ± 0.1 g/L, which is ∼10 times less than the positive control ascorbic acid (Table 2).

**TABLE 2:** Total polyphenol content and chemical antioxidant capacity of *Hypericum lanceolatum* aqueous extract (results are expressed as means ± SEM of 3 independent experiments). Note that the DPPH chemical test was done to check for potential antioxidant compounds but is of no pharmacological relevance.

	Total polyphenol content (mg GAE/g dried plant)	Chemical antioxidant capacity IC_50_-like values (g/L)
*Hypericum lanceolatum*	103.2 ± 6.1	0.86 ± 0.10
Ascorbic acid	—	0.08 ± 0.01

**FIGURE 1:**
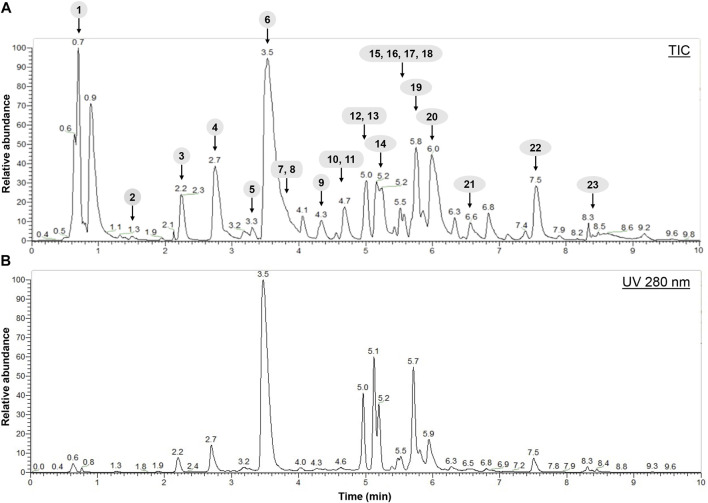
Spectra obtained after characterization of *H. lanceolatum* aqueous extract by LC-MS/MS. **(A)** Representative total ion chromatogram (TIC) obtained in negative mode **(B)** HPLC-UV chromatogram obtained at 280 nm.

In a second step, the *H. lanceolatum* aqueous extract was analyzed for its polyphenolic composition by LC-MS/MS analysis. The results revealed that *H. lanceolatum* is indeed rich in a wide variety of polyphenols, including phenolic acids and flavonols. LC-MS/MS allowed the identification of 23 polyphenolic compounds, in comparison with standards and databases (Figure 1). Among the main polyphenols identified, we observed the presence of 5-caffeoylquinic acid (chlorogenic acid), quercetin glycosides and kaempferol glycosides (see Table 3). Quantification showed a high content in caffeoylquinic acid isomers (83.4 mg/g dried plant), representing almost 65% of the total compounds identified.

**TABLE 3:** Identification and quantification (mg/g of dried plant) of compounds in *H. lanceolatum* aqueous extract by LC-MS/MS. Results are expressed as mean in mg/g. RT, retention time. Nq, not quantified.

Number	RT (min)	Compound	[M-H]^-^	mg/g
1	0.7	Quinic acid	191.0561	Nq
2	1.3	Gallic acid	169.0142	0.1
3	2.2	Protocatechuic acid	153.0193	1.0
4	2.8	3-Caffeoylquinic acid	353.0878	5.2
5	3.3	Coumaroylquinic acid isomer	337.0929	Nq
6	3.5	5-Caffeoylquinic acid (Chlorogenic acid)	353.0878	78.2
7	3.7	Caffeic acid	179.035	0.2
8	3.9	Epicatechin	289.0718	Nq
9	4.3	Coumaroylquinic acid isomer	337.0929	Nq
10	4.6	Coumaric acid	163.0401	Nq
11	4.7	Feruoylquinic acid	367.1035	Nq
12	4.9	Procyanidin B	577.1357	Nq
13	5	Quercetin 3-O-rutinoside (Rutin)	609.1461	1.5
14	5.2	Quercetin 3-O-galactoside	463.0882	4.0
15	5.4	Kaempferol-hexose-rhamnoside	593.1526	0.1
16	5.5	Isorhamnetin 3-O-glucoside 7-O-rhamnoside	623.1618	Nq
17	5.6	Quercetin 3-O-arabinoside	433.0776	0.3
18	5.7	Anthocyanins	477.1039	Nq
19	5.8	Kaempferol hexoside	447.0933	3.1
20	6	Dicaffeoylquinic acid isomer	515.1195	6.5
21	6.6	Dicaffeoylquinic acid isomer	515.1195	5.2
22	7.5	Quercetin	301.0354	0.1
23	8.4	Isorhamnetin	315.051	Nq

### *In vivo* Acute Toxicity Assay of the Aqueous Extract of *H. lanceolatum* in Zebrafish Eleutheroembryo

**FIGURE 2:**
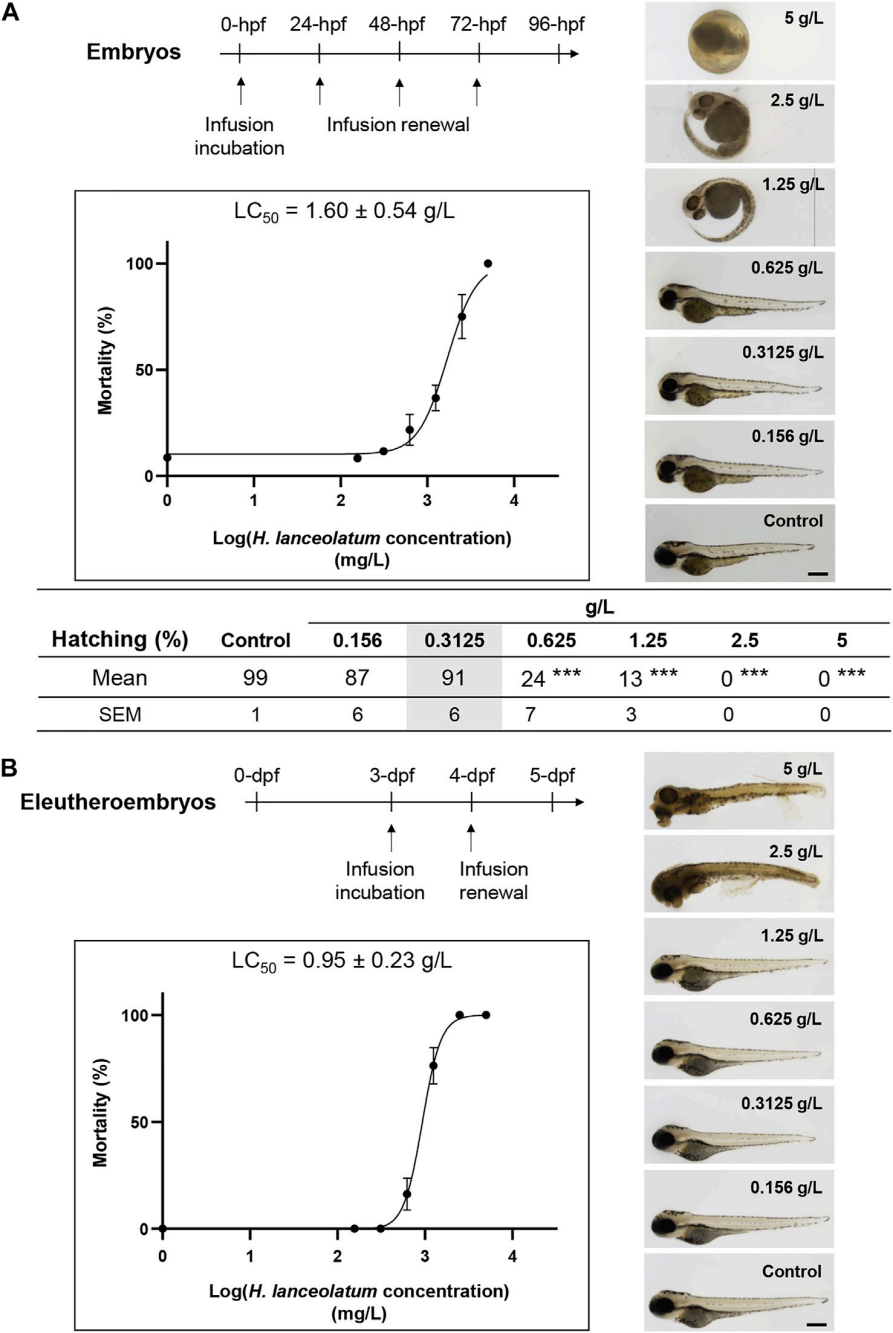
*In vivo* acute toxicity assays in zebrafish embryos and eleutheroembryos. Toxicity curves, estimated median lethal concentrations (LC_50_) and representative pictures of **(A)** 96-hpf embryos and **(B)** 5-dpf eleutheroembryos exposed to different concentrations of *H. lanceolatum* aqueous extract or E3 medium (Control). Curve values are expressed as means ± SEM (n = 60 animals/group from 3 independent experiments). ***p < 0.001 (Student t-test). Bar = 350 µm. Note that in **(A)**, at 5 g/L all the embryos are dead at 24-hpf. For 2.5 and 1.25 g/L around 26 and 63% of eleutheroembryos were alive at 96-hpf, respectively. The shaded area represents the MNTC.

To investigate the toxicity of *H. lanceolatum* aqueous extract, the Fish Embryo Acute Toxicity (FET) test was performed on newly fertilized embryos incubated with different concentrations of *H. lanceolatum* aqueous extract from 0 to 96-hpf (Figure 2A, Top scheme). Every 24 h, the eggs were carefully checked for coagulation, somite formation, tail detachment and heartbeat as indicators of abnormal development and lethality. As shown in Figure 2A, at 5 g/L, all eleutheroembryos were coagulated (mortality rate = 100%). At 2.5 and 1.25 g/L, eleutheroembryos showed some malformations such as pericardiac edema (3.75 ± 2.5%, not shown). At lower concentrations (≤0.625 g/L), no developmental alterations were observed. Interestingly, from 0.625 to 2.5 g/L, we noticed a significant proportion of unhatched eleutheroembryos. However, this parameter was not considered as a toxicity criterion according to the OECD guidelines. Furthermore, the concentration of *H. lanceolatum* aqueous extract leading to 50% of zebrafish embryo lethality (LC50) was 1.60 ± 0.54 g/L.

We also decided to develop another toxicity test on 3- to 5-dpf zebrafish to exclude embryonic toxicity. Indeed, morphogenesis and organogenesis are almost complete at these developmental stages and eleutheroembryos are able to metabolize, detoxify or excrete some toxins and other exogenous substances (Field et al., 2003; Drummond and Davidson, 2016). We consequently treated 3-dpf eleutheroembryos for 2 consecutive days and analyzed their survival and morphological defects. Briefly, from 0.156 to 1.25 g/L, treated eleutheroembryos have the same morphology as the controls. A significant mortality was observed for the highest concentrations (5–0.625 g/L) and the toxicity assay provided us a LC50 of 0.95 ± 0.23 g/L (Figure 2B) that was not significantly different from the OECD test.

In both tests, no morphological malformations (i.e., spinal curvature, pericardial edema, pigmentation, and delayed/altered development including hatching) were observed in both embryos and eleutheroembryos at a concentration of 0.3125 g/L. Based on these results, this concentration was defined as the maximum non-toxic concentration (MNTC) and was subsequently used for the rest of our study.

**FIGURE 3:**
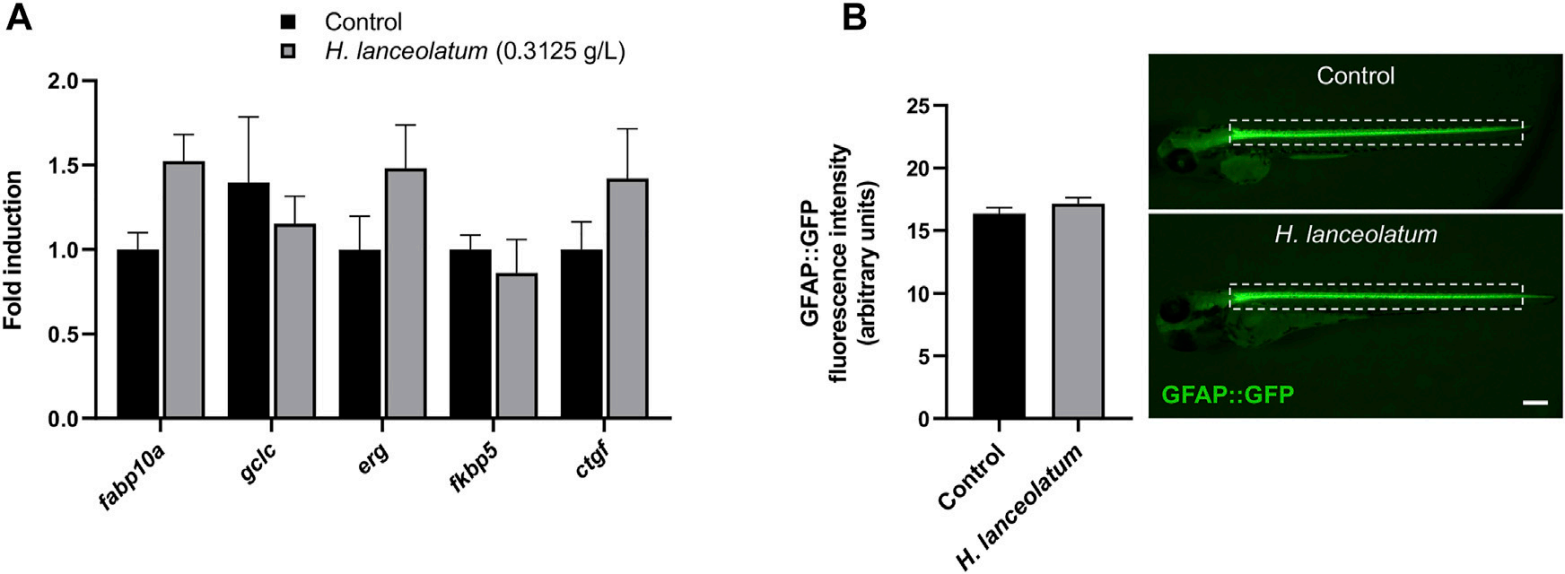
*H. lanceolatum* did not impact the expression of toxicity and endocrine disruption biomarkers. **(A)** qPCR analysis of recognized biomarkers for hepatotoxicity (*fabp10a*, *gclc*), cardiotoxicity (*erg*), nephrotoxicity (*ctgf*) and glucocorticoid signaling (*fkpb5*) of eleutheroembryos treated with *H. lanceolatum* aqueous extract at the MNTC (0.3125 g/L) or E3 medium (Control) for 2 days (n = 7–8 pools of 20 eleutheroembryos/group). **(B)** Fluorescence quantification of spinal cord in tg (*GFAP::GFP*) eleutheroembryos (dotted rectangle) and representative pictures of 48 h-treated eleutheroembryos (n = 35/group from 3 independent experiments). Results are expressed as means ± SEM. Bar = 200 µm.

In order to reinforce the non-toxic effect of *H. lanceolatum* at the MNTC, we decided to further analyze its effects on key genes of hepatotoxicity (*fabp10a*, *gclc*), cardiotoxicity (*erg*) and nephrotoxicity (*ctgf*) (Liu et al., 2018; Bauer et al., 2021) as well as *fkbp5*, a gene known to be up-regulated by glucocorticoids (Binder, 2009). To this aim, zebrafish eleutheroembryos were incubated from 3- to 5-dpf and processed for RT-qPCR analyses. Our results showed that the treatment for 2 days with the MNTC did not result in significant induction of markers for hepatotoxicity, cardiotoxicity, fibrosis and endocrine disruption (Figure 3A). In parallel, the *H. lanceolatum* extract was also studied on transgenic tg(*GFAP::GFP*) eleutheroembryos, in which the GFP is expressed in neural stem cells (Lam et al., 2009). As shown, we did not observe any change in GFP fluorescence quantification at the spinal cord level, after the incubation with *H. lanceolatum* extract (Figure 3B).

**FIGURE 4:**
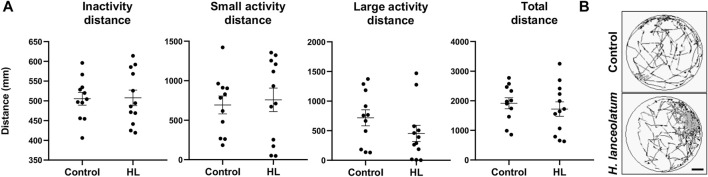
*H. lanceolatum* aqueous extract did not impact the behavioral activity in larvae. **(A)** Graphs showed the inactivity, small activity, large activity and total distances of 6-dpf larvae after 3 days of incubation with the *H. lanceolatum* aqueous extract at the MNTC (HL) or E3 medium (Control) **(B)** Total distance of control and treated larvae. Individual values were represented with means ± SEM (*n* = 11–12 animals/group). Bar = 2.3 mm.

As a last proof of concept for the lack of toxicity for the defined MNTC, we monitored a potential effect of the aqueous extract on the locomotor behavior. Because up to 6-dpf, larvae are less active (Colwill and Creton, 2011), we incubated 3-dpf eleutheroembryos for 3 days with the aqueous extract and examined the locomotor activity at 6-dpf. No changes in the inactivity, small activity, large activity and total distances traveled by control and treated larvae were observed (Figure 4). This result suggested the lack of neurobehavioral impact of the MNTC.

Taken together, these results showed that at 0.3125 g/L, the aqueous extract of *H. lanceolatum* has no obvious toxic effects on viability, expression of recognized toxicity markers and locomotion. Consequently, we decided to use this concentration for assessing its biological activities on the different models developed in this study.

### The Aqueous Extract of *H. lanceolatum* Down-Regulates GFP Expression in tg*(cyp19a1b:GFP)* Eleutheroembryos

**FIGURE 5:**
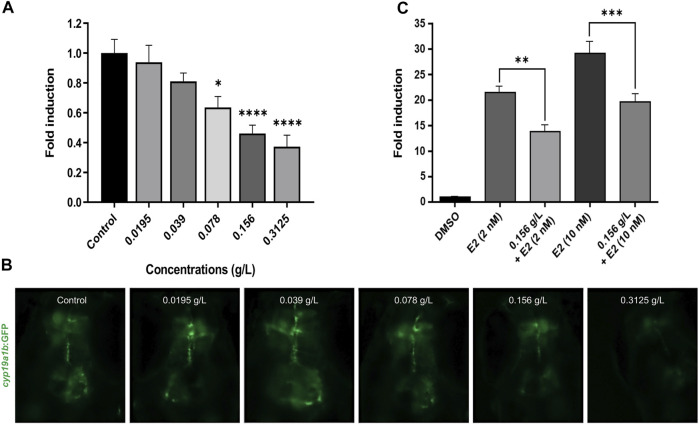
*H. lanceolatum* aqueous extract possesses an anti-estrogenic activity. **(A)** Quantification of GFP fluorescence and **(B)** representative pictures of embryos exposed to water (Control) and different concentrations of *H. lanceolatum* aqueous extract (g/L) for 96 h (n = 26–38/group from 2 independent experiments). * denotes significant differences as compared to the control group (Dunn’s multiple comparison test, *p = 0.0438 and ****p < 0.0001). **(C)** Fluorescence quantification of GFP in embryos treated with control solvent (DMSO) and E2 at two different concentrations (2 and 10 nM) alone or co-exposed with *H. lanceolatum* aqueous extract 0.156 g/L (*n* = 19–21/group). * denotes significant differences between groups (Dunnett’s multiple comparison test, **p = 0.0011 and ***p = 0.0002). All the results are expressed as fold induction above control (means ± SEM).

Considering the phenolic composition of *H. lanceolatum* and knowing that many polyphenolic compounds (i.e., flavonoids, stilbenes, lignans) could be considered as phytoestrogens (Cos et al., 2003), we evaluated whether *H. lanceolatum* extract could interfere with estrogen signaling. For that, we used the recently developed EASZY test under the OECD guidelines (OCDE, 2021) in which transgenic tg*(cyp19a1b:GFP)* zebrafish embryos are used. Expression of the *cyp19a1b* gene is well known to be up-regulated by (xeno)-estrogens through a nuclear estrogen receptor (ER)-dependent mechanisms (Menuet et al., 2005; Mouriec et al., 2009; Brion et al., 2012). Our results show that *H. lanceolatum* aqueous extract down-regulated the basal GFP expression, in a concentration-dependent manner while a strong up-regulation was observed in the positive control (EE2 0.05 nM, data not shown) demonstrating the validity of the test (Figure 5A). To further explore the negative interference of the aqueous extract of *H. lanceolatum* on *cyp19a1b* expression, a co-exposure experiment was performed with estradiol, the natural ER ligand. As expected, the embryonic estrogenic stimulation with estradiol (E2, at 2 and 10 nM) induced a strong expression of GFP as compared to the solvent control (22 to 29-fold induction respectively). The co-exposure with *H. lanceolatum* and E2 (2 and 10 nM) significantly reduced the E2-induced GFP expression (Figure 5C) thereby confirming the capacity of *H. lanceolatum* aqueous extract to negatively interfere with the ER-dependent expression of the *cyp19a1b* gene. The precise mechanism of this anti-estrogenic effect is not currently known (i.e., binding of phenolic compounds to ER as antagonists and/or activation of signaling pathway(s) leading to a negative crosstalk with the ER signaling).

### The Aqueous Extract of *H. lanceolatum* Modulates Oxidative Stress and Inflammation *in vivo*

To the best of our knowledge, there are no data documenting the antioxidant and anti-inflammatory effects of *H. lanceolatum in vivo*. In order to ascertain these properties in physiological conditions, we tested the impact of *H. lanceolatum* treatment on a tail injury model in zebrafish.

**FIGURE 6:**
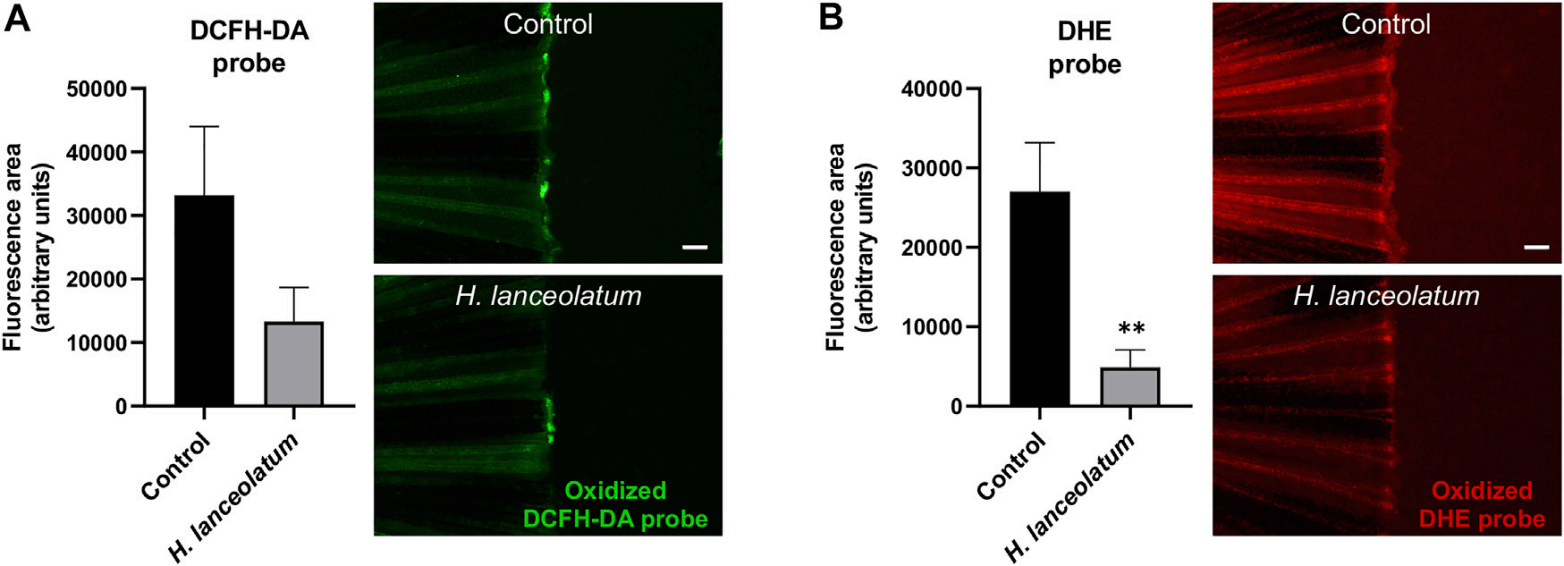
*H. lanceolatum* aqueous extract exhibits preventive antioxidant effect after caudal fin amputation. Fluorescence quantification and representative pictures of oxidized **(A)** DCFH-DA and **(B)** DHE probes after caudal fin amputation of adult zebrafish pre-incubated in water (Control) or *H. lanceolatum* aqueous extract at the MNTC (0.3125 g/L *H. lanceolatum*). Results are expressed as means ± SEM (*n* = 9/group from 3 independent experiments). **p < 0.01 (Student t-test). Bar = 200 µm.

First, in order to test the effects of *H. lanceolatum* aqueous extract on oxidative stress, we performed a caudal fin amputation in adult zebrafish and used the oxidative stress probes DCFH-DA and DHE that become fluorescent after their oxidation by hydrogen peroxide and superoxide anion, respectively. In contrast to uninjured fish, the amputated tail was strongly stained with the fluorescent oxidized probes (data not shown). The therapeutic treatment (immediately after the amputation), did not modulate oxidative stress (data not shown). We consequently examined the preventive antioxidant properties of *H. lanceolatum* extract by pretreating the fish for 4 h before tail injury. As clearly evidenced, our results showed a consistent decrease in the fluorescence of oxidized DHE and DCFH-DA probes reflecting the antioxidant properties of the *H. lanceolatum* extract (Figure 6).

**FIGURE 7:**
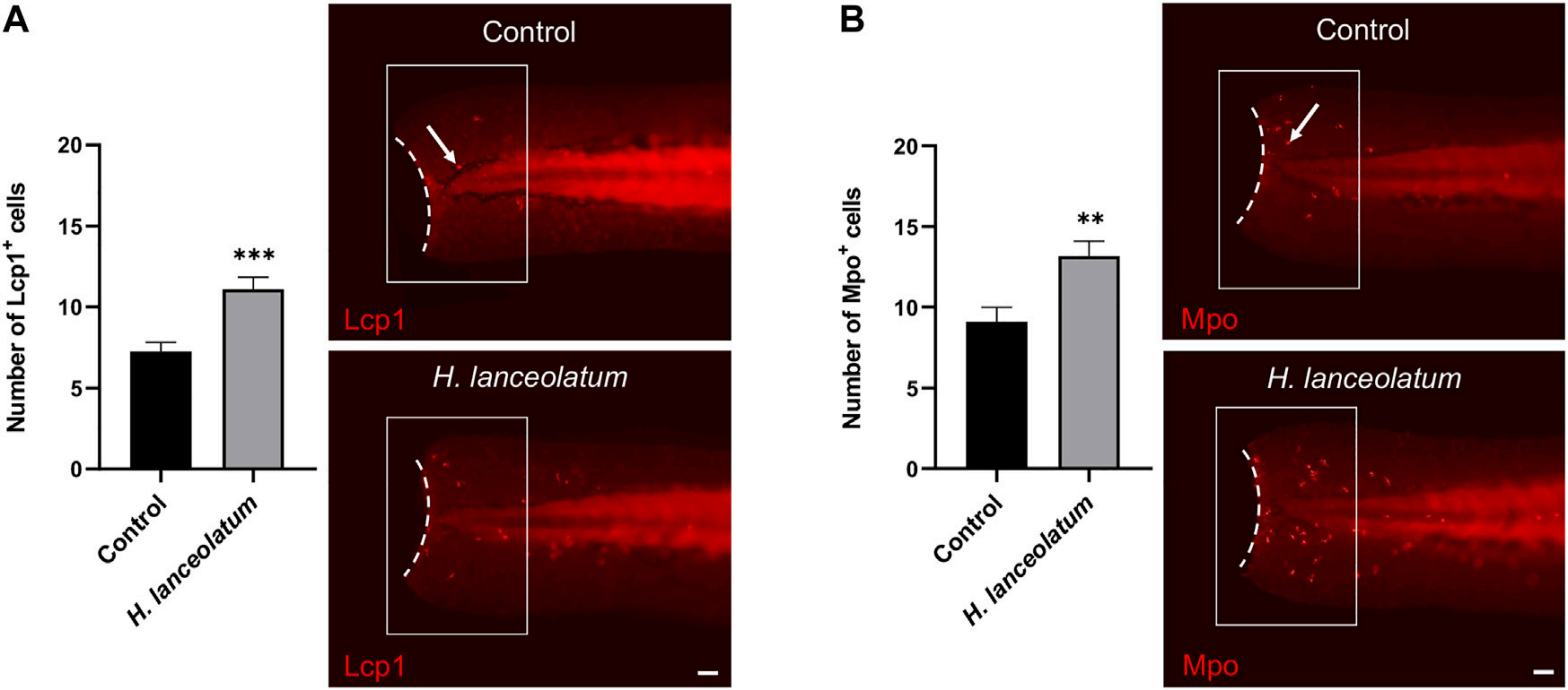
*H. lanceolatum* aqueous extract improves the recruitment of neutrophils and macrophages after tail injury. Number of **(A)** Lcp1-positive (macrophage) and **(B)** Mpo-positive (neutrophils) cells (arrows) at the injury site (white box) and representative pictures of tail amputations of 3-dpf eleutheroembryos, incubated with E3 medium (Control) or *H. lanceolatum* aqueous extract at 0.3125 g/L (*H. lanceolatum*) for 6 h. Dotted lines represent the tail amputation level. Results are expressed as means ± SEM (*n* = 34/group from 3 independent experiments). **p < 0.01, ***p < 0.001 (Student t-test). Bar = 40 µm.

In a similar way, we investigated the therapeutic effects of *H. lanceolatum* extract on immune cell recruitment, which occurs at the early phase of inflammatory response. Unfortunately, due to technical issues, we were unable to label immune cells in adult tail following injury. Consequently, to achieve this aim, we reproduced a tail injury model in 3-dpf eleutheroembryos (Miskolci and Squirrell, 2019). After tail amputation, eleutheroembryos were treated with *H. lanceolatum* infusion for 6 h and monitored for the recruitment of neutrophils and macrophages at the damaged site by performing Mpo and Lcp1 immunodetection, respectively (Morley, 2012; Aratani, 2018). We demonstrated that *H. lanceolatum* significantly increased the number of both Mpo- and Lcp1-positive cells at the injury site (Figure 7).

Overall, our results show that the aqueous extract of *H. lanceolatum* improves the antioxidant response and immune cell infiltration at the injury site. These data consequently highlight on the preventive antioxidant effects of the aqueous extract of *H. lanceolatum* and its capacity to modulate inflammatory response.

### The Aqueous Extract of *H. lanceolatum* Promotes Tail Regeneration

Oxidative stress and inflammation are well known to be key factors initiating regenerative processes in mammals and zebrafish (Schäfer and Werner, 2008; Koh and DiPietro, 2011; Dunnill et al., 2017; Nguyen-Chi et al., 2017; Paredes et al., 2021). Since caudal fin amputation in zebrafish has become a valuable model for better understanding tissue regenerative processes (Lebedeva et al., 2020), we decided to use this model to monitor the potential regenerative properties of *H. lanceolatum*.

**FIGURE 8:**
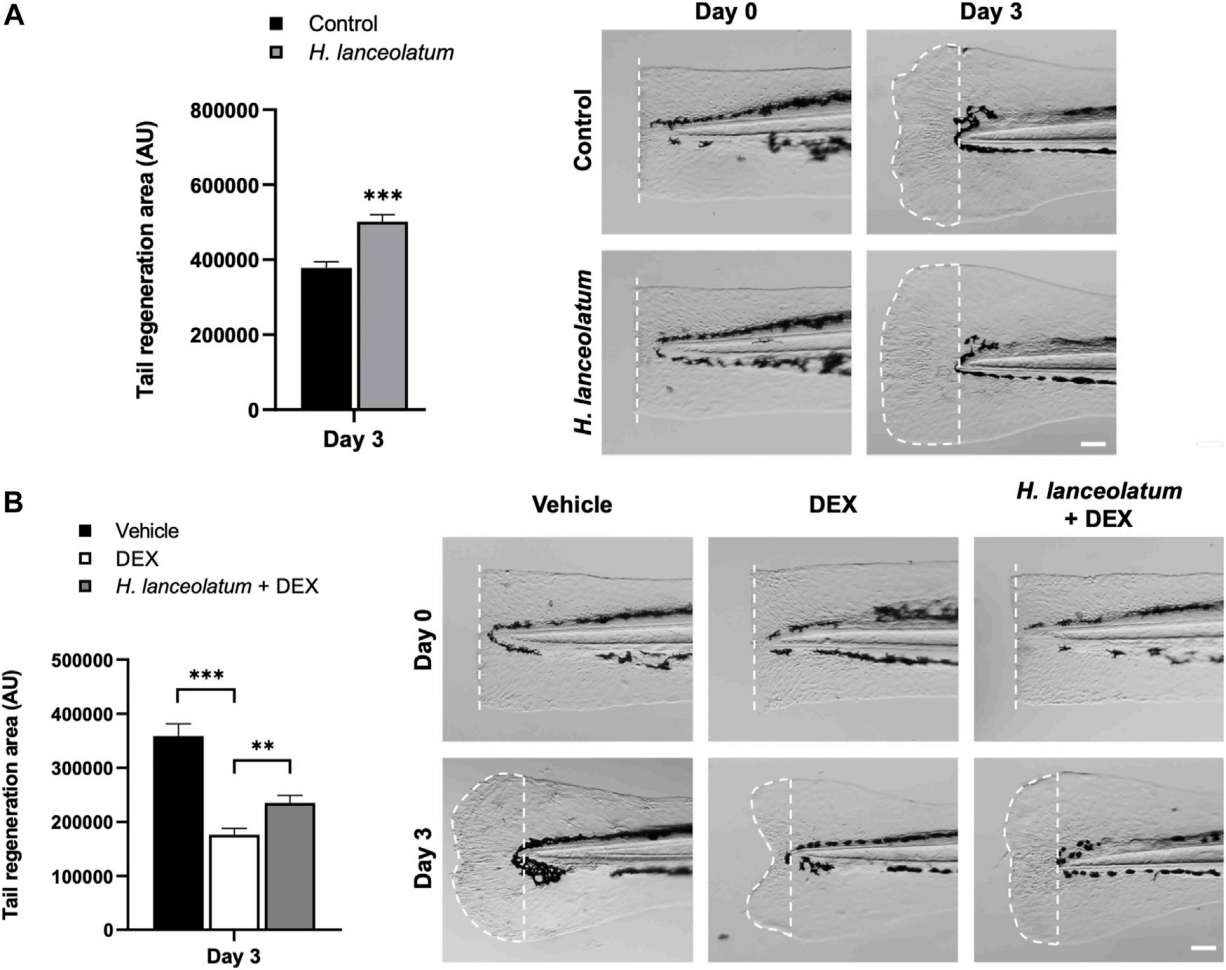
*H. lanceolatum* aqueous extract improves tail regeneration in zebrafish eleutheroembryos. **(A)** Measurement of tail regeneration area (in arbitrary units) and representative pictures of eleutheroembryos incubated in E3 medium (Control) or *H. lanceolatum* aqueous extract at 0.3125 g/L (*H. lanceolatum*), 0- and 3-days post-amputation (dotted) of 3-dpf eleutheroembryos. **(B)** Measurement of tail regeneration area and representative pictures of eleutheroembryos treated with the vehicle (0.4% ethanol), dexamethasone (500µM, DEX) or *H. lanceolatum* aqueous extract + dexamethasone (*H. lanceolatum* + DEX), 0- and 3-days post-amputation. Results are expressed as means ± SEM (*n* = 20–39/group from 3 independent experiments). **p < 0.01, ***p < 0.001 (Student t-test). Bar = 25 µm.

As a first step, we investigated the effects of *H. lanceolatum* on 3-dpf eleutheroembryos after tail amputation and monitored the regeneration at 3-days post-amputation. As shown in Figure 8A, the plant infusion exhibits pro-regenerative properties at day 3 post-amputation. In parallel, we focused on the role of inflammation in regenerative mechanisms by incubating amputated 3-dpf eleutheroembryos with the anti-inflammatory drug dexamethasone (500 µM), in the presence or not of *H. lanceolatum*. Dexamethasone inhibited the tail regeneration (Figure 8B), demonstrating the importance of inflammation in regenerative processes. In the presence of *H. lanceolatum* (*H. lanceolatum* + DEX), tail regeneration was improved compared to dexamethasone alone, strongly suggesting that *H. lanceolatum* is a modulator of inflammatory processes.

**FIGURE 9:**
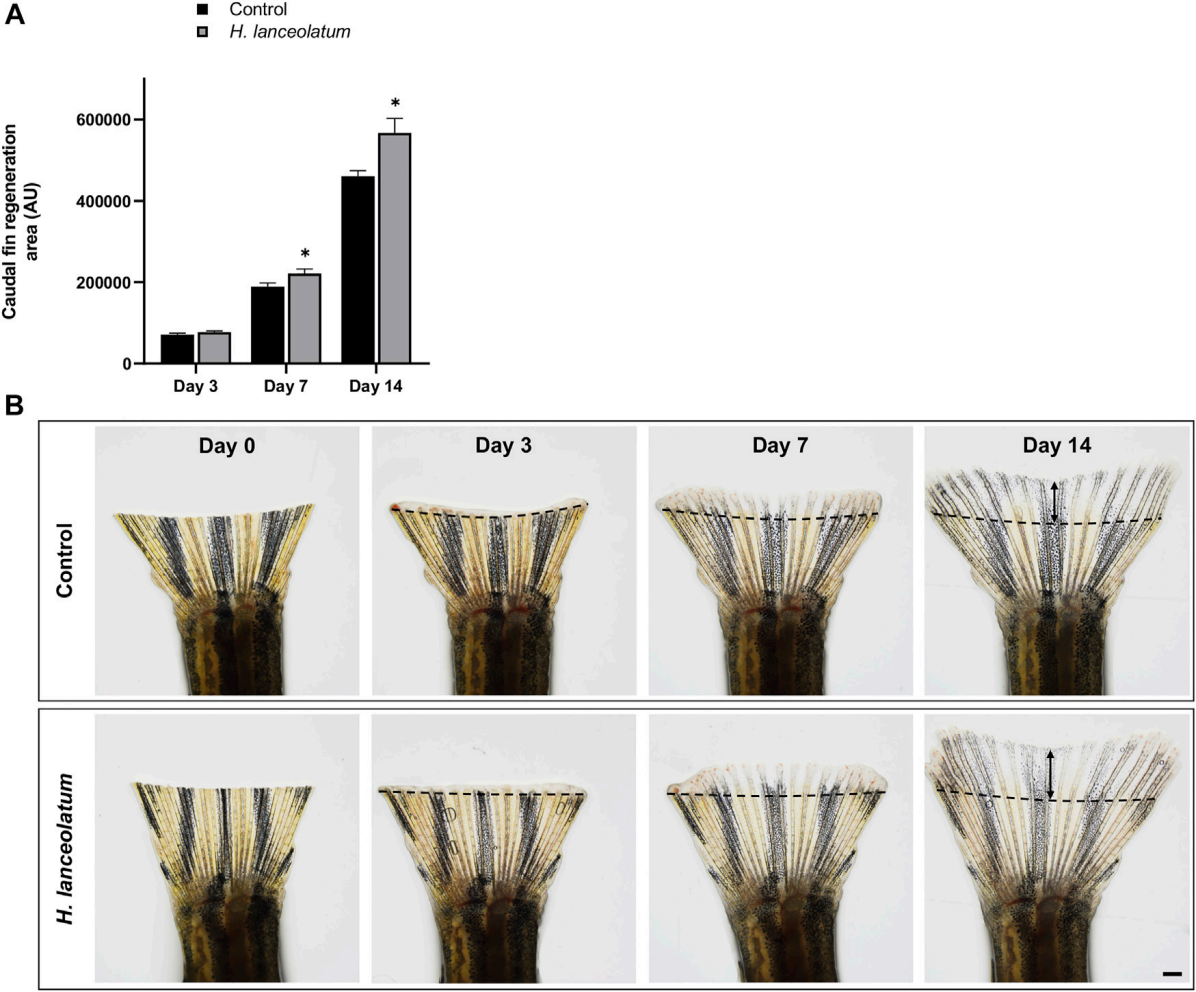
*H. lanceolatum* aqueous extract displays pro-regenerative properties in the tail of adult fish. **(A)** Measurement of tail regeneration area of adult fish after a 14-days incubation period with fish water (Control) or *H. lanceolatum* aqueous extract at 0.3125 g/L (*H. lanceolatum*). **(B)** Representative pictures of caudal fin regeneration 3-, 7-, and 14-days post-amputation. Dotted lines represent the tail amputation level. Results are expressed as means ± SEM (n = 4–7 animals/group). *p < 0.05, ***p < 0.001 (Student t-test). Bar = 625 µm.

Similar pro-regenerative effects were also observed in adults after caudal fin amputation at 7- and 14-days after injury (Figure 9). Overall, we observed that *H. lanceolatum* infusion significantly improved caudal fin regeneration compared to control amputated fish from day 7 onwards.

In summary, our results revealed that the aqueous extract of *H. lanceolatum* exerts beneficial effects on tissue regeneration, promoting an antioxidant effect and stimulating inflammatory responses, leading to accelerated tail regeneration.

### *H. lanceolatum* Displays Beneficial Effects on Pathological Disorders Related to Metabolic Diseases

In a next step, we investigated *H. lanceolatum* potential effects on zebrafish models of metabolic disorders. For that, we performed high-fat diet/diet-induced obesity models as well as chronic hyperglycemia model, as previously described (Capiotti et al., 2014; Dorsemans et al., 2017b; Ghaddar et al., 2020), in order to analyze the effects of the *H. lanceolatum* aqueous extract on metabolic disorders-related complications.

**FIGURE 10:**
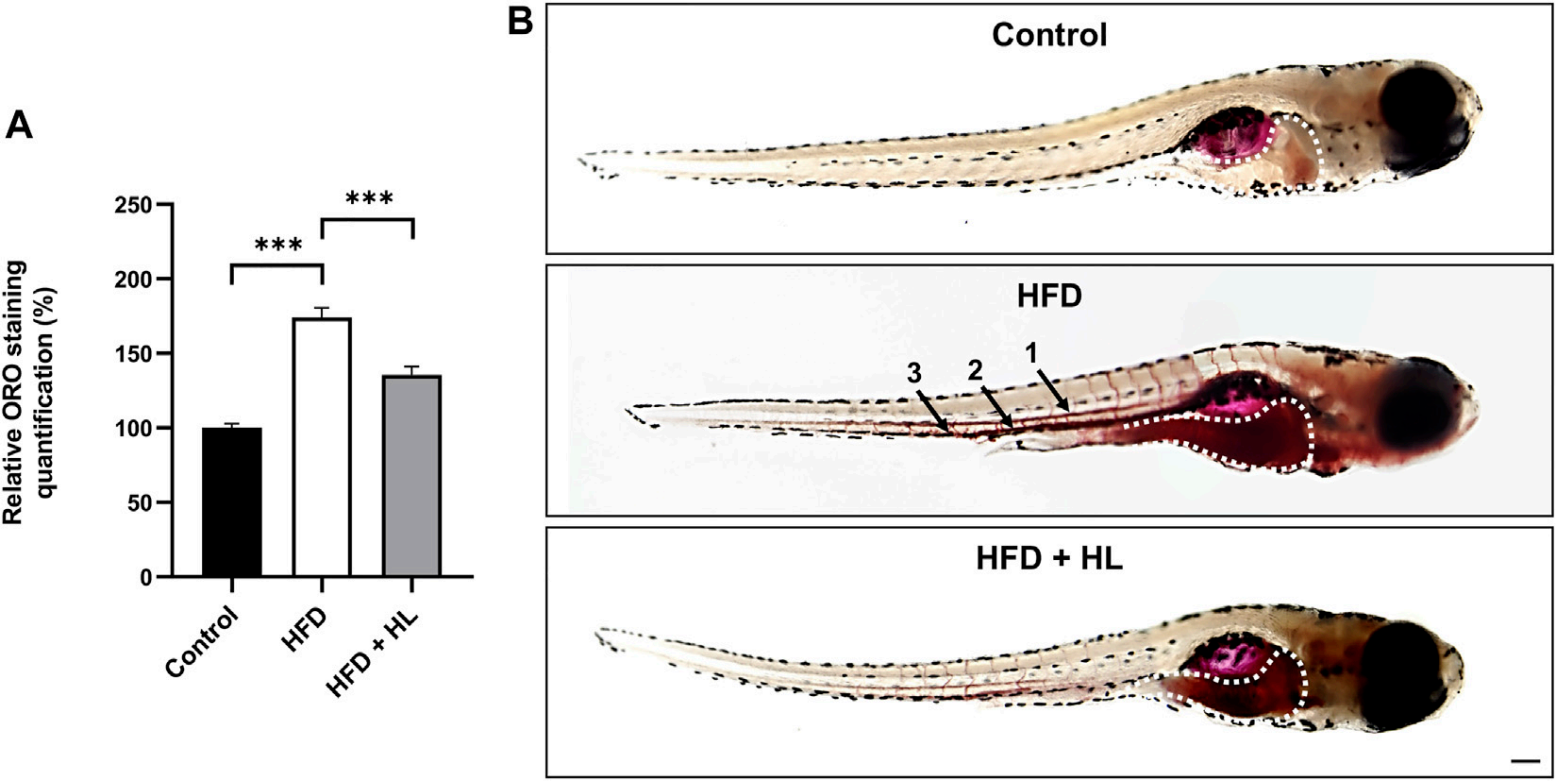
*H. lanceolatum* aqueous extract inhibits lipid accumulation in a HFD model of larvae. **(A)** Quantification of ORO staining and **(B)** representative pictures of normally fed larvae (Control), overfed (HFD) and overfed larvae treated with *H. lanceolatum* aqueous extract at 0.3125 g/L (HFD + HL). Dotted lines indicate the ORO staining in the digestive tract. Arrows show the staining in vessels including dorsal aorta (1), posterior cardinal vein (2) and caudal vein (3). Results are expressed as means ± SEM (*n* = 45 larvae/group from 3 independent experiments). ***p < 0.001 (One-way ANOVA). Bar = 150 µm.

As shown in Figure 10, larvae fed with the egg-yolk based diet (HFD) showed a higher Oil Red O (ORO) staining, reflecting an accumulation of neutral lipids and esterified cholesterol, in the digestive tract and vessels including dorsal aorta, posterior cardinal vein and caudal vein. Interestingly, larvae incubated with *H. lanceolatum* extract exhibited a significant decrease in ORO staining in relative to overfed larvae.

**FIGURE 11:**
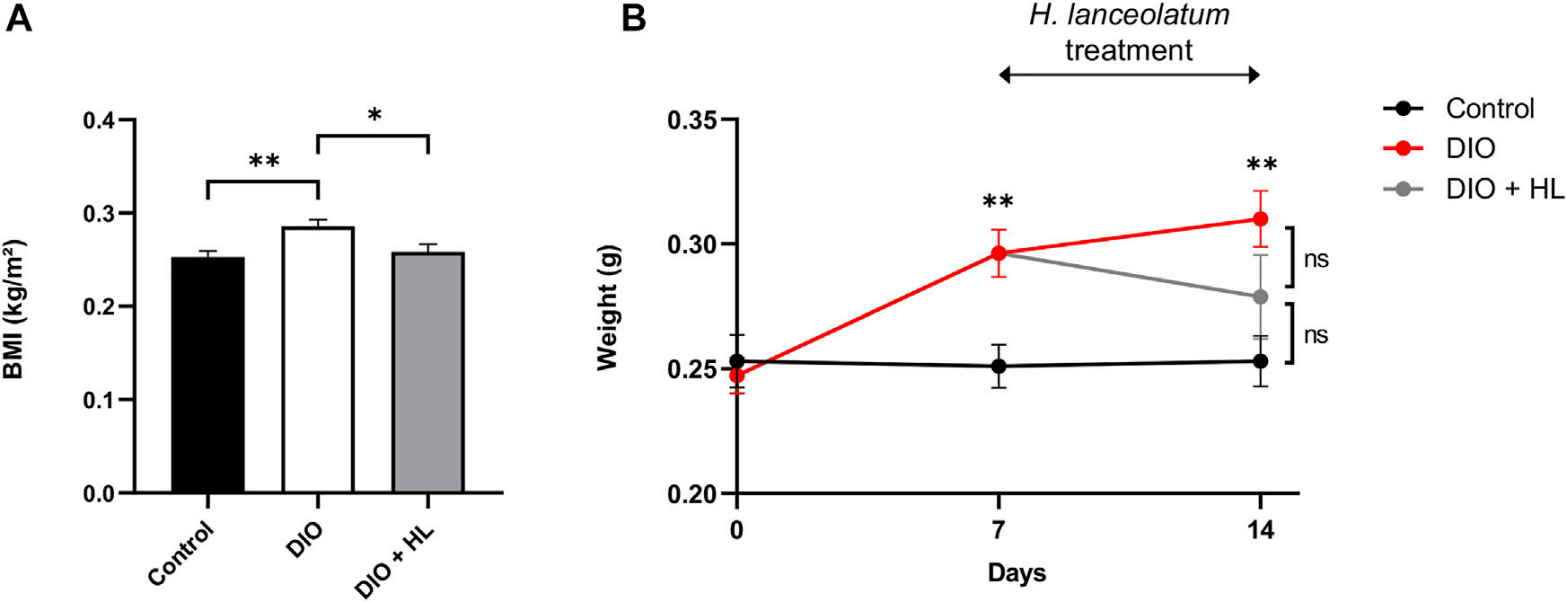
*H. lanceolatum* aqueous extract induces weight loss properties in DIO model of adult fish. **(A)** Body mass index (BMI) at day-14 and **(B)** body weight of normally fed (Control), overfed (DIO) and overfed fish treated with *H. lanceolatum* (DIO + HL). Results are expressed as means ± SEM (*n* = 8–10/group). *p < 0.05, **p < 0.01 (One-way ANOVA).

To further verify the effect of the plant extract on gain weight and associated parameters (BMI), we performed a DIO model in adult fish by overfeeding fish with dry food and *Artemia* for 14 days. After 1 week of diet, the body weight and BMI of DIO fish were significantly increased compared to controls (Figure 11). We consequently subdivided the DIO group into two groups: one overfed for 7 additional days, and another overfed for 7 additional days and treated with *H. lanceolatum* overnight. The treatment with *H. lanceolatum* aqueous extract significantly reduced the BMI (Figure 11A) and seemed to decrease the body weight (Figure 11B). Taken together, these data indicated that *H. lanceolatum* aqueous extract displayed “anti-obesity” properties.

**FIGURE 12:**
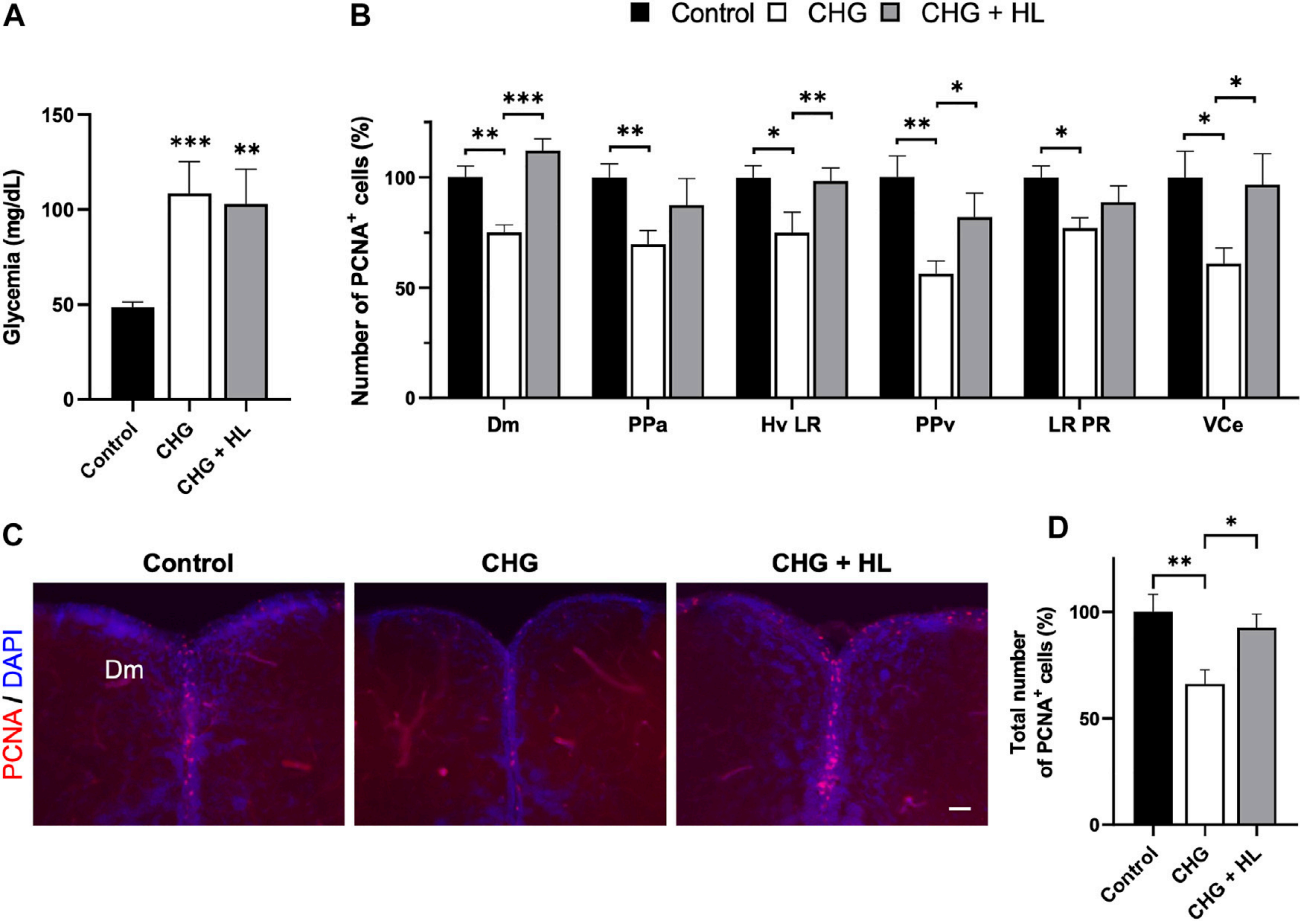
*H. lanceolatum* aqueous extract has “anti-diabetic” effects by maintaining homeostatic neurogenesis in chronic hyperglycemic adult fish. **(A)** Fasting blood glucose levels of control (Control), chronic hyperglycemic (CHG) and chronic hyperglycemic fish treated with *H. lanceolatum* (CHG + HL). **(B)** Number of PCNA-positive cells according to the studied neurogenic regions. **(C)** Representative pictures of proliferative activity (PCNA) in zebrafish brains. **(D)** Total number of PCNA-positive cells, counted in all the studied regions, in Control, CHG and CHG + HL-treated fish. Dm, medial zone of dorsal telencephalic area. PPa, parvocellular preoptic nucleus, anterior part. Hv, ventral zone of periventricular hypothalamus. LR, lateral recess of diencephalic ventricle. PPv, periventricular pretectal nucleus, ventral part. PR, posterior recess of diencephalic ventricle. VCe, valvula cerebelli. Results are expressed as means ± SEM (n = 11–14/group). *p < 0.05, **p < 0.01, ***p < 0.001 (One-way ANOVA). Bar = 40 µm.

Finally, we next investigated the effect of *H. lanceolatum* on a chronic hyperglycemia model by immersing the fish in 111 mM of glucose for 14 days. This protocol resulted in increased glycemia that was not counteracted by *H. lanceolatum* treatment (Figure 12A). Given that chronic hyperglycemia has been shown to impair neurogenesis in zebrafish (Dorsemans et al., 2017b), we decided to analyze neural stem cell (NSC) proliferation in key neurogenic niches by performing PCNA immunostaining. As expected, chronic hyperglycemia resulted in a lower number of PCNA-positive cells in the dorsomedial telencephalon (Dm), the anterior part of the preoptic area (PPa), the periventricular pretectal nucleus (PPv), two caudal hypothalamic regions (Hv LR and LR PR) and the valvula of cerebellum (VCe) compared to control fish (Figures 12B-D). Interestingly, *H. lanceolatum* treatment prevented this decreased neurogenesis, after 1 week of treatment.

## Discussion

In Reunion Island, *H. lanceolatum* is widely used in traditional medicine for treating many disorders on the basis of its antioxidant, anti-inflammatory and “anti-diabetic” properties (Lavergne, 2001). Therefore, this medicinal plant appears to be a powerful alternative to counter most of the complications associated with metabolic disorders including obesity and diabetes. However, very little was known about its potential toxicity and there was no experimental evidence on the real beneficial effects *in vivo*. In our study, we determined the Maximum Non-Toxic Concentration (MNTC) of the aqueous extract of *H. lanceolatum*, its polyphenol content by LC-MS/MS and showed its anti-estrogenic activity. We also demonstrated that *H. lanceolatum* extract, at its MNTC, displays interesting properties *in vivo* regarding immune cell recruitment, oxidative stress and regenerative processes in a zebrafish (larva and adult) model of tail amputation. Finally, we showed that *H. lanceolatum* aqueous extract has also interesting beneficial properties on metabolic disorders. Indeed, the plant extract reduces lipid accumulation in the liver and blood vessels and counteracts weight gain/BMI in overfeeding zebrafish models. In addition, it favors brain plasticity (neurogenesis) which is impaired in our hyperglycemic zebrafish model.

### *H. lanceolatum* Toxicity and Polyphenol Contents

In the present work, we first investigated the toxicity of the aqueous extract of the plant *in vivo* on zebrafish embryos, using a reliable OECD-validated toxicity test (OCDE, 2013), and also in eleutheroembryo to exclude developmental toxicity. We determined median lethal concentrations (LC_50_) for embryos and eleutheroembryo (1.60 ± 0.54 and 0.95 ± 0.23 g/L, respectively). This acute toxicity assay provided us the MNTC of 0.3125 g/L. Along with the OECD tests, we confirmed that this concentration did not impact the expression of key genes involved in hepatic, cardiac and renal functions as well as in stress hormone signaling (Liu et al., 2018; Bauer et al., 2021).

Plant toxicity can be related to the effects of many molecules including polyphenols at high concentrations (Lambert et al., 2007). Our *H. lanceolatum* aqueous extract displays an important polyphenol content (103.2 ± 6.1 mg GAE/g). Another study also described the higher polyphenol concentration of *H. lanceolatum* compared with other medicinal plants from Reunion Island (Checkouri et al., 2020). We showed the presence of caffeic acid derivatives (caffeoylquinic acid and dicaffeoylquinic acid) and flavonoids (quercetin and kaempferol) by high resolution-mass spectrometry. These data confirmed recent phytochemical analyses of aqueous and methanolic extracts from different *Hypericum* species (*Hypericum calycinum* L., *H. confertum* Choisy and *H. perforatum* L.) that also showed the presence of chlorogenic acid and quercetin as the most abundant compounds (Ersoy et al., 2019; Checkouri et al., 2020). Although the presence of 5-caffeoylquinic acid, dicaffeoylquinic acid and quercetin glycosides may explain the antioxidant and anti-inflammatory effects observed at the MNTC, the toxicity observed at higher concentrations in our study could be due to the high content of these molecules. Indeed, some studies demonstrated that high concentration of chlorogenic acid (7 mg/kg/day) and quercetin (1900 mg/kg/day), the two main components of our *H. lanceolatum* aqueous extract, could have toxic effects in rats (Dunnick and Hailey, 1992; Du et al., 2013). As well, we should consider the strong toxic effects of alkaloids and pyrrolizidine alkaloids (including developmental, hepato-, genotoxicity and carcinogenicity) resulting from frequent consumption of teas and infusions (Manteiga et al., 1997; Wiedenfeld, 2011; Knutsen et al., 2017; Oketch-Rabah et al., 2020).

### *H. lanceolatum* Displays Anti-estrogenic Activity

Compounds of plant origin including some polyphenols (such as genistein and daidzein which are abundant in soy, or resveratrol) are able to disrupt estrogen signaling through pro- and anti-estrogenic activities due to their capacity to bind to nuclear estrogen receptors (Cos et al., 2003). Such endocrine disruption has already been demonstrated *in vitro* for chlorogenic acid, quercetin and kaempferol (Resende et al., 2013; Hsu et al., 2021). Interestingly, phytoestrogens can be associated with many health benefits including the reduction of menopausal symptoms or a lowered risk to develop cardiovascular diseases, osteoporosis and cancers (Patisaul and Jefferson, 2010). However, it can also be harmful for human health, promoting hormone-sensitive cancers or perturbing reproductive physiology (Chandrareddy et al., 2008; Liang and Shang, 2013). Here, we reported the anti-estrogenic activity of the aqueous extract of *H. lanceolatum* which involves a decrease in the expression of estrogen-synthetizing enzyme (aromatase B encodes by *cyp19a1b* gene). These anti-estrogenic properties reinforce the fact that traditionally, *H. lanceolatum* is not recommended for pregnant women because of its effects on female reproductive system (i.e. abortive/menstruation).

### *H. lanceolatum* Modulates Oxidative Stress, Immune Cell Recruitment, and Regeneration

In traditional medicine, *H. lanceolatum* is used as a modulator of the oxidative and inflammatory processes (Lavergne, 2001). Some *in vitro* studies also confirm the antioxidant effects of *H. lanceolatum* extracts on red blood cells and preadipocytes (Poullain et al., 2004; Checkouri et al., 2020). In the present study, we investigated the effects of the aqueous extract of *H. lanceolatum* using a caudal fin amputation model. Indeed, zebrafish tail amputation is known to generate both oxidative stress and inflammation (Morales and Allende, 2019). Our results revealed the preventive antioxidant capacity of *H. lanceolatum* extract after tail amputation, but the therapeutic treatment was inefficient. This difference may be explained by the activation of the antioxidant defenses including antioxidant enzymes throughout *H. lanceolatum* pretreatment, prior to injury (Paredes et al., 2021). In parallel, we monitored the recruitment of macrophages and neutrophils at the site of injury 6 h after the tail sectioning. At this time point, the number of immune cells is known to reach a peak at the injured site (Miskolci and Squirrell, 2019). Interestingly, the number of macrophages and neutrophils was significantly higher for *H. lanceolatum*-treated eleutheroembryo than for controls. Given that the recruitment of immune cells is essential for initiating tissue regeneration in both fish and mammals (Schäfer and Werner, 2008; Koh and DiPietro, 2011), we investigated the effects of *H. lanceolatum* aqueous extract on tail regeneration and demonstrated its pro-regenerative properties in both eleutheroembryo and adult zebrafish. We further confirmed the beneficial role of inflammation in regeneration through dexamethasone treatment, an immunosuppressive glucocorticoid. As previously reported, tail regeneration was reduced in dexamethasone-treated larvae (Sharif et al., 2015) but was improved in larvae treated with dexamethasone + *H. lanceolatum*. Considering that it has been suggested that glucocorticoids may alter the migration of immune cells at the injury site (Chatzopoulou et al., 2016), we suggested that *H. lanceolatum* may prevent this inhibition by promoting neutrophil and macrophage recruitment and then, allowing the tissue repair. Overall, these data support the key roles of oxidative stress and inflammation in tissue repair and demonstrate the beneficial effect of *H. lanceolatum* in regenerative processes. Such a positive impact on regeneration could be supported by the presence of 5-caffeoylquinic acid (chlorogenic acid isomer), quercetin and kaempferol. Indeed, these polyphenols have been shown to stimulate wound healing in rats by improving the redox status and inflammation and/or promoting angiogenesis and proliferation of epithelial and fibroblastic cells (Bagdas et al., 2015; Gopalakrishnan et al., 2016; Kant et al., 2017; Özay et al., 2019).

### *H. lanceolatum* Improves Metabolic Parameters and Limits Neurogenesis Dysfunction Induced by Obesity and Diabetes

It is well established that recurrent consumption of polyphenols is inversely correlated with the incidence of diabetes, obesity, cardiovascular diseases and cancers (Zamora-Ros et al., 2016; Guo et al., 2017; Del Bo et al., 2019). To test the potential “anti-obesity” and “anti-diabetic” effects of *H. lanceolatum*, we used our previously developed overfeeding and hyperglycemia models to mimic the complications associated with metabolic disorders (Capiotti et al., 2014; Zhou et al., 2015; Dorsemans et al., 2017a; Dorsemans et al., 2017b; Ghaddar et al., 2020).

Feeding zebrafish larvae with egg yolk powder resulted in the accumulation of neutral lipids and esterified cholesterol in the digestive tract and blood vessels, which is consistent with another study (Zhou et al., 2015). In adults, overfeeding induced a significant body weight gain, as previously shown (Oka et al., 2010; Montalbano et al., 2016; Ghaddar et al., 2020). In our models, *H. lanceolatum* therapeutic treatment significantly reduced the lipid accumulation in larvae and the BMI in overfed adult fish. The underlying mechanisms can be related to a decreased feeding behavior, a reduced lipid uptake or a greater lipid catabolism. It has been reported that *H. perforatum* L. aqueous extract (which has the same plant taxonomy as *H. lanceolatum*) significantly reduced serum levels of triglycerides, LDL-C (low density lipoprotein cholesterol) as well as oxidative stress biomarkers induced by hyperlipidemia in rats (Zou et al., 2005; Ghosian Moghaddam et al., 2016). In addition, supplementation with chlorogenic acid and quercetin has been shown to modulate the expression of some lipogenesis- and lipolysis-related genes, respectively, in mice and chicken after a HFD (Wang et al., 2019; Wang et al., 2021). The authors also suggested that the prevention of obesity-related disturbances might be closely associated with the modulation of the gut microbiota (Wang et al., 2019; Wang et al., 2021). Together with these reports, our findings support the contribution of *H. lanceolatum* functional components decreasing lipid accumulation and improving BMI. However, the precise mechanism underlying this hypolipidemic activity requires further investigations. It is nevertheless important to consider that zebrafish are ectothermic animals and are not able of thermogenic-mediated processes (Den Broeder et al., 2015). In addition, some metabolic processes including those associated to the leptin signaling pathways are less conserved and could impact the disease phenotypes (Zang et al., 2018).

Finally, we studied the “anti-diabetic” properties of *H. lanceolatum* using a hyperglycemic model (Capiotti et al., 2014; Dorsemans et al., 2017a). Treatment with *H. lanceolatum* had no impact on blood glucose levels but, interestingly, prevented a decrease in neural stem cell proliferation. In diabetes, high glucose levels contribute to several brain dysfunctions, including impaired neural plasticity, blood-brain barrier integrity, and neurogenic processes that can lead to cognitive and memory decline (Lang et al., 2009; Ho et al., 2013; Ramos-Rodriguez et al., 2014; Dorsemans et al., 2017a). The interest of *H. lanceolatum* in preventing neurological complications can be supported by the actions of its bioactive molecules (Ito et al., 2008; Wu et al., 2016). However, this interesting result should be confirmed in a preclinical model of diabetes using for instance Db/Db or HFD mice.

### Zebrafish Concerns Regarding Extrapolation to Humans

In the present work, we used zebrafish for investigating the toxicity and potential preventive/therapeutic effects of *H. lanceolatum*. Zebrafish is a recognized alternative model for studying physiological processes linked to human diseases, considering the strong genomic homology and the well-conserved physio-pathological mechanisms with mammals (Oka et al., 2010; Howe et al., 2013). Thus, zebrafish clearly helps for screening a wide variety of potent pharmacological molecules as well as toxicants. However, despite these advantages, it is important to consider the limitations of the zebrafish model. The absence of placenta during embryogenesis and the fact that fish are poikilothermic animals implie that drugs could be metabolized with different rate compared to mammals. As well, the way of administration of the extract should be taken into consideration (immersion versus intravenous/intraperitoneal treatment). Regarding metabolic disorders, the strong adaptability and regenerative properties of zebrafish could have a significant differential impact on the processes investigated compared to mammals. So far, the use of zebrafish is a first step to validate some potential effects by further studies in well-validated preclinical rodent models.

## Conclusion

In conclusion, this work reported for the first time the toxicity of the aqueous extract of *H. lanceolatum* using reliable zebrafish toxicity tests. The analysis of the composition of *H. lanceolatum* extract identified phenolic acids (chlorogenic acid isomers) and flavonoids (quercetin, kaempferol derivatives) as major compounds in the aqueous extract. Here we demonstrated that *H. lanceolatum*, used in traditional Reunionese medicine, has strong antioxidant, pro-regenerative, anti-lipid accumulation, and neurogenic properties on zebrafish at its MNTC. In addition, our results suggest the potential effect of *H. lanceolatum* aqueous extract to counteract diabetes- and obesity-related complications. The present study is a first step providing evidence for non-toxic and potential therapeutic effects of *H. lanceolatum*. Further investigations are required to elucidate the mechanisms underlying the activities of traditional medicinal plants in the prevention and/or treatment of metabolic disorders using preclinical rodent models. Finally, it is important to consider the anti-estrogenic effect of *H. lanceolatum* in case of pregnancy or estrogen-dependent diseases.

## Data Availability

The original contributions presented in the study are included in the article/Supplementary Material, further inquiries can be directed to the corresponding authors.
